# Drug-related problems reported by patients with rheumatic diseases: an observational study

**DOI:** 10.1186/s41927-023-00326-x

**Published:** 2023-04-18

**Authors:** Lex L. Haegens, Victor J. B. Huiskes, Elisabeth M. Smale, Charlotte L. Bekker, Bart J. F. van den Bemt

**Affiliations:** 1 Department of Rheumatology, Sint Maartenskliniek, Ubbergen, Gelderland The Netherlands; 2 Department of Pharmacy, Sint Maartenskliniek, Ubbergen, Gelderland The Netherlands; 3 Department of Pharmacy, Radboudumc, Nijmegen, Gelderland The Netherlands

**Keywords:** Rheumatic diseases, Drug-related problems, Observational, Patient-reported

## Abstract

**Background:**

Drug-related problems can negatively influence treatment outcome and well-being for patients with rheumatic diseases. Thus, it is important to support patients in preventing or resolving drug-related problems as quickly as possible. To effectively develop interventions for this purpose, knowledge on the frequency and character of drug-related problems is needed. Therefore, this study aims to quantify and characterize drug-related problems reported by patients with inflammatory rheumatic diseases along their treatment process.

**Methods:**

A prospective observational study was conducted in a Dutch outpatient pharmacy. Adult patients with rheumatic diseases that were prescribed medication by a rheumatologist were questioned about experienced DRPs by telephone 4 times in 8 weeks using a structured interview-guide. Patient-reported DRPs were scored on uniqueness (i.e., if a specific DRP was reported in multiple interviews by one individual, this was counted as one unique DRP) and were categorized using a classification for patient-reported DRPs and analysed descriptively.

**Results:**

In total, 52 participants (median age 68 years (interquartile range (IQR) 62–74), 52% male) completed 192 interviews with 45 (87%) participants completing all 4 interviews. The majority of patients (65%) were diagnosed with rheumatoid arthritis. Patients reported a median number of 3 (IQR 2–5) unique DRPs during interview 1. In subsequent interviews, patients reported median numbers of 1 (IQR 0–2), 1 (IQR 0–2) and 0 (IQR 0–1) unique DRPs for interviews 2–4 respectively. Participants reported a median number of 5 (IQR 3–9) unique DRPs over all completed interviews. Unique patient-reported DRPs were most frequently categorized into (suspected) side effects (28%), medication management (e.g., medication administering or adherence) (26%), medication concerns (e.g., concerns regarding long-term side-effects or effectiveness) (19%) and medication effectiveness (17%).

**Conclusions:**

Patients with rheumatic diseases report various unique DRPs with intervals as short as two weeks. These patients might therefore benefit from more continuous support in-between contact moments with their healthcare provider.

**Supplementary Information:**

The online version contains supplementary material available at 10.1186/s41927-023-00326-x.

## Background

Pharmacotherapy is the cornerstone of the treatment of many inflammatory rheumatic diseases. Drugs such as non-steroidal anti-inflammatory drugs (NSAIDs) can relieve pain and disease-modifying anti-rheumatic drugs (DMARDs) can moreover decrease disease activity and disability, and even decrease or prevent joint damage in inflammatory rheumatic diseases like rheumatoid arthritis [1]. For optimal treatment outcomes and patient safety, it is essential that medication is used as agreed upon between patient and healthcare provider.

However, many studies have demonstrated that medication use in inflammatory rheumatic diseases is suboptimal: only half of the patients take their medication as prescribed (i.e., are adherent to their long-term therapy) [2], the majority of patients suffers from at least one side effect from their medication [3], more than 90% of patients have concerns about their medication [3] and more than 90% of the biological DMARDs are not stored at the right temperature at home [4]. These drug-related problems (DRPs) could have clinical consequences such as higher disease activity and more side effects, which eventually could lead to an increase in morbidity, mortality and healthcare costs [5–7].

As many inflammatory rheumatic diseases require chronic pharmacotherapy, it is conceivable that this population is at risk for experiencing DRPs at any given point during their treatment. However, in general patients only visit their healthcare provider up to four times a year. In between these consultations patients could benefit from additional support when experiencing DRPs to improve treatment outcomes and increase medication safety. Knowledge regarding frequency and nature of DRPs that patients experience is needed to determine whether patients are in need of additional support. Existing research into DRPs within rheumatic diseases focuses on cross-sectional assessment of DRPs from a healthcare provider’s perspective (e.g., using chart-based medication reviews) [8, 9]. However, as each patient is different, the possible need for additional support is patient-specific. Thus, a more patient-centred approach of assessing DRPs could be more desirable. Additionally, research shows that it is essential to include the patient perspective when identifying clinically relevant DRPs [10, 11].

Therefore, this study aims to quantify and characterize drug-related problems reported by patients with inflammatory rheumatic diseases along their treatment process.

## Methods

### Study design and setting

A longitudinal observational study with structured interviews was performed among patients from the rheumatology department of the Sint Maartenskliniek in the Netherlands between December 2019 and April 2020. This study was performed in accordance with the Declaration of Helsinki. The study was approved by the Medical Ethics Review Committee of the Radboud university medical centre Nijmegen, the Netherlands, with protocol reference number 2021–7505.

Patients were eligible for participation if they were at least 18 years of age and received medication prescribed by a rheumatologist from the outpatient pharmacy. Eligible patients who received medication in the two weeks prior to study start were ordered randomly using Excel and invited for participation by telephone. Patients were included after verbal informed consent was obtained and patients indicated availability for four consecutive interviews, after which the first interview was immediately conducted. In total, each participant was interviewed four times with two weeks in-between each interview to reflect a period shorter than the interval in-between patients’ consultations with their healthcare provider, which usually take place every three to six months. Inclusion continued until 50 patients agreed to participate.

### Outcomes

Primary outcome was the number of unique DRPs (i.e., DRPs that were not reported earlier during the study by an individual patient) reported per patient per interview and per patient for all interviews combined. Secondary outcomes included the type and subtype of unique DRPs reported per interview and for all interviews combined.

### Data collection

Pharmacy technicians conducted structured interviews by telephone using a questionnaire aimed at identifying DRPs related to the medication prescribed by a rheumatologist. Pharmacy technicians received a half-day training in conducting structured interviews for the purpose of this study. For each interview, the same questionnaire was used. Each interview was conducted without reference to when reported DRPs were experienced and without reference to previous interviews from individual patients. Thus existence of reported DRPs in earlier interviews was not actively assessed by the interviewer to ensure the reporting of all relevant DRPs by participants. Uniqueness of DRPs was assessed afterwards during data processing by two researchers independently (LH, VH) after which discrepancies were discussed and resolved. When deemed necessary by the interviewing pharmacy technician, counselling on reported DRPs was offered per normal pharmacy practice. This counselling lies outside the scope of the current study. The questionnaire (English translation available in Additional file 1) was developed for the purpose of this study and was constructed after (1) an extensive search for relevant literature on existing medication management support services (e.g., frameworks, questionnaires, and tools to identify or classify DRPs) [12–18] combined with the expertise of the authors, (2) formulating and selecting questions with the aim of identifying all types of DRPs that patients may experience by one author (VH) and (3) checking of formulation and completeness of the questionnaire by a second author (BB). Participants’ answers were reported on a data collection form by the pharmacy technician during the interview. In addition to experienced DRPs, patient demographics including sex, age, diagnosis, disease duration, and a medication overview at the time of the interview were recorded.

### Data processing

After data collection, reported DRPs were categorized using a classification system. Existing classification systems for DRPs mainly focus on DRPs from a healthcare provider’s perspective, aimed at identifying causes of DRPs within the healthcare provider’s medication process (e.g., prescription and dispensing). These classifications lacked classes representing the patient perspective sufficiently for the aim of this study. Therefore, two pharmacists (BB & VH) constructed a classification system based on a selection of relevant classes from existing classification systems supplemented with additional classes to better represent possible DRPs that patients experience along their patient journey [19, 20]. This resulted in a seven-type classification system (Table 1).

**Table 1:** Classification for drug-related problems

Type	Description
(Suspected) side effects	All problems regarding undesired effects that occur when using medication. Suspected side effects (i.e., when the cause of a side effect might be related to medication for a patient’s rheumatic disease) also fall under this type.
Medication management	All problems regarding opening medication packaging, administering and storing medication, travelling with medication, and medication adherence.
Medication concerns	All problems regarding concerns and fears related to medication use. Includes concerns about long-term effectiveness, long-term side effects and toxicity, among others.
Medication effectiveness	All problems regarding the experience of health problems related to the underlying disease (i.e., pain, inflammation, etc.) that are currently under- or overtreated. Problems regarding effectiveness and dosage reduction also fall under this type.
Information needs	All problems in which a patient explicitly requests for additional information, for example regarding long-term effects or mechanism of action for a specific drug.
Contra-indications	All problems regarding contra-indications that prevent a patient from using their drug(s) as intended.
Logistics	All problems regarding delivery, brand or brand changes of medication, and pharmacy service.

First, each DRP was scored on uniqueness within patients by determining if a DRP had been reported in earlier interviews by the same individual, with only unique DRPs being considered for analysis. Next, reported DRPs were categorized using the constructed classification system. Each unique DRP was exclusively classified to a single type of DRPs to prevent duplicate counting. This categorization and determination of uniqueness within patients was conducted by three researchers (LH, LMS & VH) independently, after which discrepancies were discussed until consensus was reached. In answers that contained multiple unique DRPs, each DRP was categorized individually.

### Statistical analysis

Data were analysed using Stata (Stata/IC 13.1 for Windows). Patient characteristics and numbers of unique DRPs were descriptively analysed. Based on the distribution of the data, either mean and standard deviation (SD) or median and interquartile range (IQR) were calculated. Missing interviews were not included in the analyses.

## Results

### Participants

Fifty-two patients were included with a median age of 68 years (IQR 62–74) and 52% were male. The majority of participants (65%) were diagnosed with rheumatoid arthritis (RA), and four participants (8%) were diagnosed with two concurrent rheumatic diseases. Participants had a median disease duration of ten years (IQR 5–20). The four most commonly used categories of medication prescribed by a rheumatologist were conventional synthetic DMARDs (65%), biological DMARDs (62%), gastric acid-reducing drugs (60%) and NSAIDs (54%). 19 patients (37%) used a combination of a conventional synthetic DMARD and a biological DMARD. Patients used a mean of 5 (SD 2) drugs prescribed by a rheumatologist. Patient characteristics are shown in Table 2.

**Table 2:** Baseline characteristics of the study population (n = 52)

Sex (male), n (%)	27 (52%)
Age (years), median (IQR)	68 (62–74)
Diagnosis, number of patients (%)	
Rheumatoid arthritis	34 (65%)
Psoriatic arthritis	7 (13%)
Spondyloarthritis	3 (6%)
Polymyalgia rheumatica	3 (6%)
Gout	3 (6%)
Osteoarthritis	2 (4%)
Osteoporosis	1 (2%)
Polyarthritis	1 (2%)
Sjogren’s syndrome	1 (2%)
Temporal arteritis	1 (2%)
Disease duration (years), median (IQR)	10 (5–20)
Medication categories prescribed by rheumatologist, number of patients (%)	
DMARDs	
csDMARDs	34 (65%)
bDMARDs	32 (62%)
Analgesics	
NSAIDs	28 (54%)
Paracetamol	26 (50%)
Corticosteroids	16 (31%)
Opioids	4 (8%)
Other	
Gastric acid-reducing drugs	31 (60%)
Folic acid	27 (52%)
Osteoporosis medication	20 (38%)
Laxatives	7 (13%)
Artificial tears	7 (13%)
Antigout preparations	2 (4%)
Raynaud’s syndrome medication	2 (4%)
Antiemetics	1 (2%)

bDMARD, biologic disease-modifying anti-rheumatic drug; csDMARD, conventional synthetic disease-modifying anti-rheumatic drug; DMARD, disease-modifying anti-rheumatic drug; IQR, interquartile range; NSAID, non-steroidal anti-inflammatory drug

### Interviews

Participants completed 192 structured interviews in total, with 45 participants (87%) completing all four interviews. Main reasons for not completing all interviews were refusal to (further) participate, therapy discontinuation and loss to follow-up.

### Drug-related problems

**Fig. 1:**
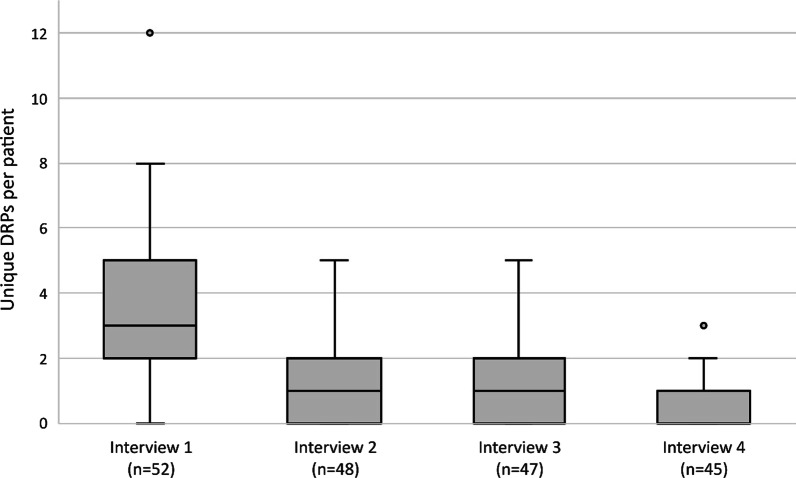
Unique drug-related problems per patient per interview. DRP, drug-related problem; Legend: ^○^, outlier (value outside 1.5 times the interquartile range above the upper quartile or below the lower quartile)

Patients reported a median of 3 (IQR 2–5) unique DRPs during the first interview (Fig. 1). The follow-up interviews resulted in median numbers of unique DRPs of 1 (IQR 0–2), 1 (IQR 0–2) and 0 (IQR 0–1) for interviews two to four respectively. Participants reported a median number of 5 (IQR 3–9) unique DRPs over all completed interviews, with 2 participants (4%) reporting no DRPs at all.

### Types of DRPs per interview

**Fig. 2:**
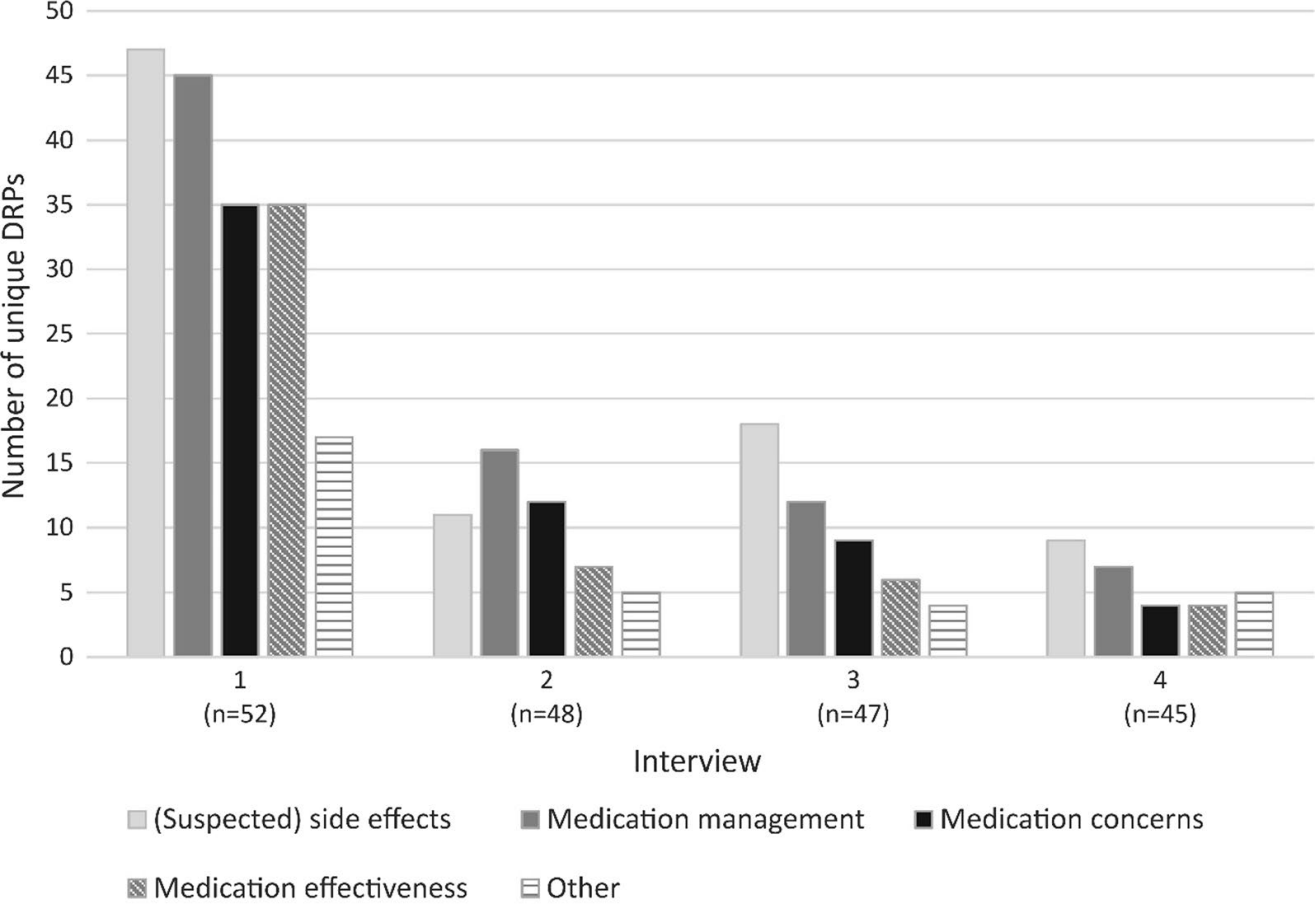
Total number of unique drug-related problems per type per interview. *Category ‘Other’ consists of types that accounted for less than 5% of the total number of unique DRPs: ‘Information needs’, ‘Contra-indications’ and ‘Logistics’

The most reported types of DRPs were (suspected) side effects, medication management, medication concerns and medication effectiveness in all four interviews. In interview 2, the order of the three most common types of unique DRPs differed from the other interviews, with (suspected) side effects being the third instead of the first most common type reported by patients. The distribution of types of unique DRPs per interview round is outlined in Fig. 2.

### Subtypes of all reported DRPs

Considering all reported DRPs, patients’ most frequently reported subtypes of DRPs (n (% of total unique DRPs)) were insufficient effect of therapy (30 (10%)), concerns regarding long-term effectiveness (19 (6%)), drug-use in general (17 (6%)), and problems with dose reduction (14 (5%)). Within the most reported type of unique DRPs regarding (suspected) side effects, a larger variety of subtypes was present than in other types, resulting in relatively infrequent subtypes within (suspected) side effects. The three most common subtypes for each type of unique DRPs are shown in Table 3.

**Table 3:** Types and top 3 subtypes of unique DRPs

Type	n (% of total)	
Subtype		n (% within type)
(Suspected) side effects	85 (28%)	
Infection(s)		7 (8%)
Fatigue		6 (7%)
Nausea		5 (6%)
Medication management	80 (26%)	
Administering injection		11 (14%)
Storage when travelling		11 (14%)
Intake schedule while travelling		11 (14%)
Medication concerns	60 (19%)	
(Long-term) effectiveness		19 (32%)
Drug-use (general)		17 (28%)
(Long-term) side-effects		8 (13%)
Medication effectiveness	52 (17%)	
Insufficient effect		30 (58%)
Dose reduction		14 (27%)
Unclear effect		6 (12%)
Information needs	13 (4%)	
(Long-term) side-effects		4 (31%)
Mechanism of action		2 (15%)
Infection(s)		1 (8%)
Contra-indications	9 (3%)	
Infection(s)		5 (56%)
Pregnancy		2 (22%)
Blood levels		1 (11%)
Logistics	9 (3%)	
Availability		5 (56%)
Service		2 (22%)
Reimbursement		1 (11%)

Number (and percentage of total) drug-related problems within each type and the three most common subtypes (and percentage within types) of drug-related problems within each type. Subtypes of drug-related problems that occur in multiple types of drug-related problems were exclusively counted in the corresponding type

## Discussion

This study quantified and characterized drug-related problems reported by patients with rheumatic diseases along their treatment process. By conducting four structured interviews in an eight-week period, this study showed that the majority (96%) of participants reported at least one unique DRP within the study period, with a median of 5 (IQR 3–9) unique DRPs over all completed interviews combined. Moreover, on average participants reported at least one new DRP during follow-up interviews two and three, with intervals of two weeks in-between interviews. The most frequently reported unique DRPs by patients were (suspected) side effects, problems with medication management (such as non-adherence or difficulties with storing or administering medication), concerns regarding medication use (such as long-term effectiveness or possible side-effects) and problems regarding medication effectiveness. Although interview two resulted in a different top three of most common types of unique DRPs when compared to interviews one, three and four, the top three subtypes per interview remained constant. This study quantified relatively high numbers of DRPs (median of 5 (IQR 3–9) unique DRPs per patient) when compared to previous studies, which reported 1–2 DRPs per patient in rheumatic diseases populations [8, 9, 21]. Main DRPs in patients with rheumatic diseases identified in other (pro- and retrospective) studies include treatment safety, treatment effectiveness, adverse reactions and drug-drug interactions. These types of DRPs were limitedly reported by our study participants. Our study mainly found DRPs regarding (suspected) side effects, medication management and medication concerns. Reasons for differences can be explained by the patient perspective adopted in this study, dissimilar study populations, or the method of data-collection.

However, it is difficult to compare our results to these previous studies, as previous studies assessed DRPs cross-sectionally using a single medication chart review from a healthcare provider’s perspective, without patient involvement. Furthermore, registering DRPs from patients likely resulted in both higher numbers of DRPs and differences in types of DRPs assessed, which can be caused by disparities between what patients and healthcare providers consider important and prioritize in treatment of a patient [22, 23].

The importance of patient-involvement in assessing DRPs is highlighted by several studies. Furthermore, Kwint et al. [11] found that 27% of all DRPs were identified during patient interviews and Kari et al. [10] concluded that 84% of the most significant DRPs in their study could only have been identified with patient involvement. Lastly, evidence exists for a positive effect of patient-focused medication reviews on patient-reported health quality [24]. Despite this importance, Huiskes et al. [25] showed that communication about DRPs between patients and healthcare providers is suboptimal with almost one in six raised DRPs not being discussed, indicating a lack of patient-orientation when assessing DRPs.

Although the eight-week study period is relatively short compared to the often chronic nature of disease and treatment for patient with rheumatic diseases, this study showed that patients reported unique DRPs with intervals as short as two weeks. This indicates that problems occur in between consultations between patient and healthcare provider, for which patients currently may not receive support.

A few methodological limitations should be acknowledged. First, DRPs were assessed using structured patient interviews. On one hand, patients might not have reported DRPs related to medication adherence or emotional or sensitive topics, which would result in an underestimate of the number of DRPs assessed. On the other hand, the number of DRPs might be overestimated due to socially desirable answers. Second, as DRPs assessed in this study were patient-reported, we could have identified DRPs that may not (yet) have had a negative impact on a patient’s health. However, one can argue that these DRPs could potentially pose barriers against using medication, which may cause harm to patients in the future. From a preventive point of view, we therefore believe these DRPs cannot be ignored. Third, patients that received medication prescribed by a rheumatologist two weeks prior to the start of this study were randomly selected and approached for participation. Nonetheless, patients that were experiencing DRPs were possibly more willing to participate than patients that did not experience DRPs at all, influencing the generalizability of our findings. Fourth, this study was conducted in a single centre, which is the largest specialized centre for rheumatic diseases in the Netherlands, which possibly limits generalizability. On one hand, patients in our population might not be comparable to the general rheumatic diseases population in the Netherlands as specialized centres tend to treat more complex patients than general hospitals. On the other hand, the higher level of usual care could result in less DRPs, although this effect is believed to be minimal as pharmacological treatment is highly standardized and protocolized across hospitals in the Netherlands.

As this study demonstrated that patients with rheumatic diseases report DRPs with intervals as short as 2 weeks, this population possibly benefits from more continuous support regarding DRPs along their treatment. Therefore, future research should focus on developing interventions to prevent or identify and resolve DRPs as quickly as possible, in addition to current contact moments between patient and healthcare provider. To effectively develop and apply such interventions, a few aspects should be studied. First, it is important to gather data on cause and duration of DRPs, as this might contain important clues about the urgency and relevance of DRPs. Second, (sub)groups of patients with increased risk of experiencing DRPs should be researched, as interventions should preferably target patients at risk of experiencing DRPs and at key moments in the patient journey that frequently cause DRPs (such as initiation of a new therapy) [26]. Third, DRPs should be prioritized on clinical relevance to determine which DRPs should be intervened on first. And fourth, patients’ needs for support should be identified to personalize this support, to ensure that developed interventions will be successfully adopted by the target population.

## Conclusion

Patients with rheumatic diseases report unique DRPs with intervals as short as two weeks, related to (suspected) side effects, problems with medication management (such as non-adherence or difficulties with storing or administering medication), concerns regarding medication use (such as long-term effectiveness or possible side-effects) and problems regarding medication effectiveness. This indicates a need for additional support with DRPs in-between consultations between patient and healthcare provider. This study represents a first step in offering optimal support of patients with rheumatic diseases regarding DRPs, by providing insight into the frequency at which this population experiences DRPs.

## Supplementary Information

**Additional file 1.** English translation of the questionnaire used for structured interviews.

## Data Availability

The datasets generated during and analysed during the current study are available from the corresponding author on reasonable request.

## Abbreviations

bDMARD: Biological disease-modifying anti-rheumatic drug
csDMARD: Conventional synthetic disease-modifying anti-rheumatic drug
DMARD: Disease-modifying anti-rheumatic drug
DRP: Drug-related problem
IQR: Interquartile range
NSAID: Non-steroidal anti-inflammatory drug
SD: Standard deviation
