# Supplementary material for: Drug-related problems reported by patients with rheumatic diseases: an observational study
Source: BMC Rheumatol. 2023 Apr 18;7:7. doi: 10.1186/s41927-023-00326-x (PMC10111673; doi:10.1186/s41927-023-00326-x)
Supplement: Supplementary file 1 — Additional file 1. English translation of the questionnaire used for structured interviews. [file 41927_2023_326_MOESM1_ESM.docx]

**Additional file 1**

English translation of questionnaire used for structured interviews

|  | **Question** |
| --- | --- |
| **1** | *How is using your rheumatic medication going in general?* |
| **2** | *What do you notice of your rheumatic medication in general?* |
| **3** | *What effect do you experience?* |
| **4** | *What side-effects do you experience?* |
| **5** | *What problems do you experience regarding drug use?* |
| **6** | *What practical problems do you experience?* |
| **7** | *What expectations do you have?* |
| **8** | *To which extent do you worry about your medication use?* |
| **9** | *Have you missed or rescheduled a dose in the last 2 weeks?* |
| **10** | *When do you experience difficulties using your medication?* |
| **11** | *What support do you experience from your surroundings in using your medication?* |
| **12** | *What questions do you have regarding rheumatic medication? What else would you like to know?* |
